# Transcutaneous Auricular Vagus Nerve Stimulation for the Treatment of Myoarthropathic Symptoms Associated With Temporomandibular Disorders—A Pilot Randomised Controlled Trial

**DOI:** 10.1111/joor.70039

**Published:** 2025-08-15

**Authors:** Lea S. Prott, Vanessa Kaldenhoven, Alfons Hugger, Robert Langner, Petra C. Gierthmuehlen, Mortimer Gierthmuehlen

**Affiliations:** ^1^ Department of Prosthodontics Medical Faculty and University Hospital Düsseldorf, Heinrich‐Heine‐University Düsseldorf Germany; ^2^ Institute of Systems Neuroscience Medical Faculty and University Hospital Düsseldorf, Heinrich‐Heine‐University Düsseldorf Germany Düsseldorf Germany; ^3^ Institute of Neuroscience and Medicine Brain and Behavior (INM‐7) Research Center Jülich Jülich Germany; ^4^ Department of Neurosurgery University Medical Center Knappschaftskrankenhaus Bochum GmbH Bochum Germany

**Keywords:** myofascial pain, neuromodulation, temporomandibular disorders, transcutaneous auricular vagus nerve stimulation, vagus nerve, vagus nerve stimulation

## Abstract

**Background:**

Transcutaneous auricular vagus nerve stimulation (taVNS) is a safe and feasible treatment for a variety of acute and chronic pain conditions. However, no evidence about taVNS effectiveness in patients with chronic pain associated with temporomandibular disorders (TMD) is available.

**Objective:**

To evaluate the feasibility of and compliance with taVNS in participants experiencing chronic TMD pain and potential effects on pain, psychological well‐being, muscle activity, and kinematics.

**Methods:**

Twenty adults with chronic TMD pain were randomised to receive taVNS (*n* = 10) or sham (*n* = 10). In the taVNS group, stimulation was performed on the left tragus for 4 h daily (25 Hz, pulse width 250 μs, 28 s on/32 s off). In the sham group, an inactive non‐functional sham electrode was used. Patient‐reported outcome measures (GCPS, PHQ‐9, GAD‐7, PHQ‐15, and OHIP‐G14), muscle activity, and kinematics were assessed at baseline, 4 weeks, and 8 weeks. Compliance was assessed using a smartphone app, which recorded daily stimulation time and intensity.

**Results:**

Recruitment and retention rates were high (100% and 90%, respectively), with 83% adherence to the intervention. Participants receiving taVNS showed a large effect on oral health‐related quality of life, and at least a small but potentially important effect on pain intensity, anxiety, depression, severity of somatic symptoms, muscle activity, and kinematics. However, none of these differences were statistically significant. No serious adverse events were identified.

**Conclusion:**

taVNS proved feasible in participants with chronic TMD pain, suggesting potential benefits for symptom management. Future studies with larger sample sizes and extended follow‐up durations are necessary to confirm efficacy and safety.

## Graphical abstract

This pilot randomised controlled trial investigated the feasibility and potential therapeutic effects of transcutaneous auricular vagus nerve stimulation (taVNS) in participants experiencing chronic pain associated with temporomandibular disorders (TMD). High recruitment and retention rates were achieved. taVNS showed a large effect on oral health‐related quality of life and at least a small but potentially important effect on several clinical outcomes (e.g., pain intensity, anxiety, muscle activity). These findings support the need for larger‐scale trials to confirm efficacy and explore long‐term outcomes.

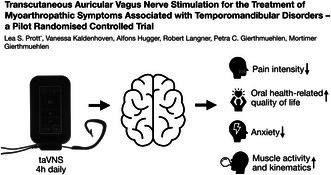

**Abbreviations**
ANOVA: Analysis of variance
CE: Certification indicating legal conformity and safety for medical devices.
CI: Confidence interval
CONSORT: Consolidated Standards of Reporting Trials extension to pilot and feasibility trials
COVID‐19: Coronavirus Disease 2019 pandemic
DC: Diagnostic Criteria (e.g., for TMD)
DGFDT: Deutsche Gesellschaft für Funktionsdiagnostik und Therapie
DRKS: Deutsches Register Klinischer Studien
EMG: Electromyography
GAD‐7: Generalised Anxiety Disorder 7‐item scale
GCP: Good Clinical Practice
GCPS: Graduation of Chronic Pain Scale
MMO: Maximum Mouth Opening
MVC: Maximum Volumetric Contraction
OHIP‐G14: Oral Health Impact Profile German 14‐item version
OHRQL: Oral Health‐Related Quality of Life
PHQ‐15 PROM: PHQ‐15=Patient Health Questionnaire 15‐item somatic symptom severity module PROM=Patient‐reported outcome measure
PHQ‐9: Patient Health Questionnaire 9‐item depression module
RCT: Randomised Controlled Trial
taVNS: Transcutaneous Auricular Vagus Nerve Stimulation
TMD: Temporomandibular Disorders
VNS: Vagus Nerve Stimulation

## 1 Introduction

Temporomandibular disorders (TMD) comprise a complex group of musculoskeletal conditions, mainly characterised by myofascial and masticatory muscle pain, as well as restricted jaw mobility [1, 2, 3]. Approximately 30% of TMD cases associated with acute pain progress to chronic pain, which is defined as symptoms persisting for 3 months or longer. Modern stress and fast‐paced lifestyles have amplified its public health impact. Nowadays, TMD ranks as the second most common chronic musculoskeletal pain condition worldwide, affecting 6%–9% of adults [4]. Chronic TMD is most prevalent in women aged between 20 and 40 [5] and is more frequent in individuals with low socioeconomic status, military veterans, and comorbid mental health conditions [6]. The COVID‐19 pandemic further exacerbated this burden through social isolation and limited access to care [7, 8], with 50% of TMD patients reporting worsened symptoms and mental health disorders increasing by over 25% [7].

Vagus nerve stimulation (VNS), initially developed as an implanted device in 1997 [9], is now also available as a non‐invasive transcutaneous auricular VNS (taVNS). It is approved by the US Food and Drug Administration (FDA) for the treatment of drug‐resistant epilepsy, depression, and cluster headaches [10, 11]. Like the implanted version, taVNS electrically stimulates the auricular vagus nerve, activating the nucleus tractus solitarii. This nucleus signals via various neural pathways to both subcortical and cortical regions, resulting in an activation of brain areas associated with inflammation, pain perception, and emotional regulation [12, 13]. Current evidence suggested that an increased sympathetic tone may exacerbate the intensity of pain experienced by TMD patients [14]. In contrast, elevated vagal activity is hypothesized to have therapeutic potential across a wide spectrum of conditions [15, 16]. TMD is frequently associated with comorbidities such as migraine and fibromyalgia [17, 18], both of which are linked to imbalances in the autonomic nervous system [19]. VNS has demonstrated efficacy in treating various acute and chronic pain disorders that either share pathophysiological similarities with TMD or commonly occur as comorbidities, including migraine, depression, and tinnitus [20].

A previous study investigated the effect of taVNS compared to sham stimulation on myofascial pain in university students, assessing electromyographic parameters, pressure pain thresholds, and anxiety [21]. The study identified a significant reduction in anxiety and a decrease in masseter muscle electromyography activity and pressure pain threshold. However, participants included in that trial did not have a prior history of chronic pain associated with TMD. Previous studies have identified taVNS as a safe and feasible treatment option for a variety of acute and chronic pain conditions, yielding promising results [10, 20, 22]. Based on these observations, taVNS may hold potential for improving symptoms of TMD. This pilot trial aims to evaluate whether taVNS is a feasible therapeutic option for individuals with chronic pain associated with TMD. The findings will inform the design and statistical methods of a subsequent large‐scale randomised controlled trial (RCT).

## 2 Materials and Methods

This study was conducted at the University Medical Center Düsseldorf (Department of Prosthodontics) in collaboration with the University Medical Center Knappschaftskrankenhaus Bochum (Department of Neurosurgery). The study protocol received approval from the Ethics Commission of the University Hospital Düsseldorf (Reference number: 2022‐1889) on July 1, 2022. Additionally, this pilot trial has been registered in the Deutsches Register Klinischer Studien (DRKS) database (DRKS00029724), and the protocol has been published previously [23] (DOI: https://doi.org/10.1186/s40814‐024‐01447‐x).

### 2.1 Study Design

This study was designed as a parallel‐arm, blinded RCT with a 1:1 allocation ratio to compare an active intervention with a sham device. A randomisation sequence was created using a computer‐generated numerical method. Concealed allocation to study arms and blinding of participants were implemented. During the first study visit, participants were assigned to one of the two groups by a researcher who was unaware of the randomisation sequence. Blinding of study participants was achieved by using an inactive sham electrode that resembled the active device. Patient‐reported outcomes (PROMs), muscle activity, and kinematic outcomes were assessed at T0 (baseline), T1 (4 weeks after T0), and T2 (4 weeks after T1). A figure of the study design, endpoints, and outcomes can be found in the protocol [23].

### 2.2 Study Participants

Participants (≥ 18 years) currently being treated for TMD associated with chronic pain (Grade III or IV of the Graduation of Chronic Pain Scale [GCPS] according to von Korff [24]) at the Department of Prosthodontics at the University Medical Center Düsseldorf were screened for eligibility (Table 1). The presence of myogenic and/or arthrogenic pain served as the primary inclusion criterion. Additional TMD diagnoses, such as disc displacement or other non‐painful conditions, were permitted, provided that patients met the core pain‐related eligibility criteria. Comorbidities commonly associated with TMD, such as neck pain and migraine, were not exclusion criteria.

**TABLE 1:** Participants' eligibility criteria.

Inclusion criteria
Chronic temporomandibular disorders (TMD) Age ≥ 18 years Provided written informed consent to participate in the trial Positive response to the question: “Do you have pain in the right side of your face, the left side, or both?” Grade III or IV of the Graduation of Chronic Pain Scale (GCPS) No or stable depression for at least 4 weeks
Exclusion criteria
Orofacial pain or diagnosis(es) that do not qualify as myalgia, myofascial pain, or arthralgia based on the Diagnostic Criteria for Temporomandibular Disorders Severe psychiatric disorder (e.g., schizophrenia) Interventions with other vagus nerve stimulation or history of vagotomy History of cardiac diseases: bradycardic arrhythmia (e.g., sick sinus syndrome), heart failure, condition after myocardial infarction Active implant, e.g., pacemaker, defibrillator, neurostimulator, cochlear implant or drug delivery device, or ventricular shunt Any inability to understand the inform consent documents Progressive neurological disease (e.g., Parkinson's disease, multiple sclerosis, epilepsy) Pregnancy Prostate carcinoma Presence of a skin condition such as infection, psoriasis, or eczema at the stimulation site Presence of an anatomical abnormality preventing insertion of the ear electrode Presence of a serious medical condition preventing study participation Acute tinnitus

A dentist, certified in DC/TMD and highly experienced in TMD diagnosis, conducted examinations in accordance with the diagnostic criteria (DC/TMD). Before enrollment, participants received a thorough explanation of the study procedures and provided written informed consent. The identification of any exclusion criteria, the onset of severe cardiac arrhythmias, or the participants' withdrawal of consent warranted discontinuation from the study. Patients were allowed to continue TMD treatments (e.g., splint therapy, physiotherapy) during the study period.

### 2.3 taVNS Intervention and Sham

**FIGURE 1:**
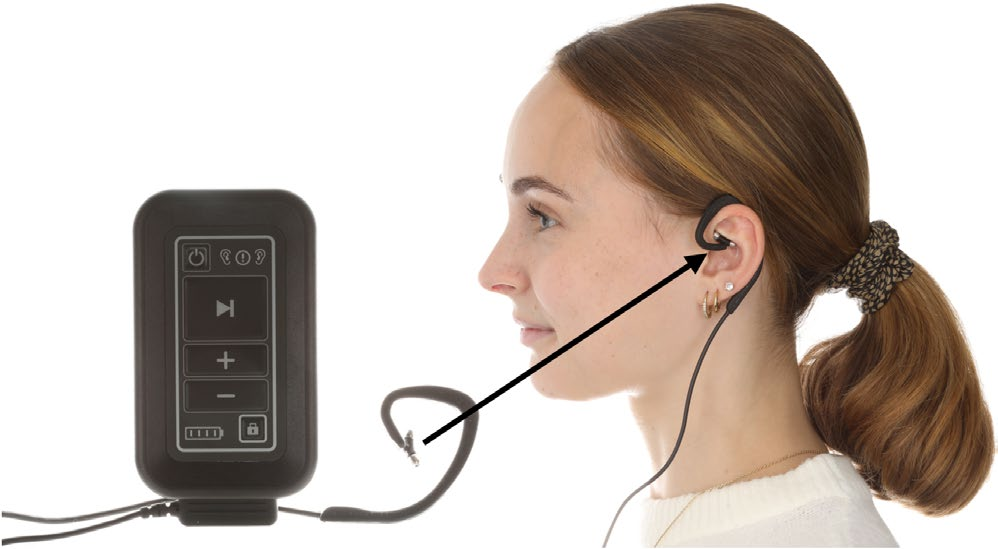
tVNS‐L device with electrode placed for stimulation at the concha of the left ear.

The tVNS‐L device (tVNS technologies, Erlangen, Germany) is intended for transcutaneous electrical stimulation of the auricular branch of the vagus nerve. It uses a headphone‐like electrode that targets the cymba conchae (an area located between cartilage grooves superior to the crus of the helix) of the left ear (Figure 1). The device received European certification, confirming its compliance with European safety and regulatory standards, in 2010 for the treatment of epilepsy and depression, in 2012 for chronic pain, and in 2019 for anxiety disorders [25]. It operates with a fixed frequency of 25 Hz and a pulse width of 250 μs, following a 28 s‐on/32 s‐off protocol. The stimulation intensity is adjustable by the participants but should remain above the perception threshold. Conductive gel (Elektroden‐Gel, C + V Pharma‐Depot GmbH) was applied to the electrode's surface to enhance conductivity. For the sham group, an inactive electrode identical in appearance to the active electrode was employed to simulate the treatment. During the initial visit, the active electrode was initially set at the stimulation threshold and subsequently adjusted to an intensity level below this threshold. Participants were instructed to adjust the intensity at this level during the study. The active electrode was then replaced with the non‐functional sham electrode to ensure no stimulation was delivered. Participants were instructed to use the device for 4 h throughout the day and directed to inform the principal investigator promptly should any side effects arise. One week after the study began, an examiner contacted the participant to address any questions or concerns that may have arisen during the initial days of use.

### 2.4 Outcomes

#### 2.4.1 Primary Outcomes

The primary outcomes were defined as the feasibility of the intervention, participants' adherence to taVNS, and the determination of the magnitude of the effect of the intervention on pain reduction, measured via the GCPS score.

Feasibility outcomes included the following: (1) Recruitment rate, defined as the proportion of participants enrolled in the study relative to the target number to recruit (*n* = 20). Recruitment was considered successful when ≥ 70% of the target (≥ 14 participants) were recruited; (2) retention rate, defined as the proportion of participants randomised to a study arm who completed all outcome measures (visits 0, 1, and 2). Retention was considered successful when at least ≥ 75% of participants completed all measures; (3) randomisation success, defined as the degree to which participants are evenly allocated to the intervention and control arm; (4) blinding success, defined as the extent to which the investigators successfully implemented blinding across visits and during interactions with participants; (5) compliance with assessment procedures included three domains: (5.1) Compliance with PROMs defined as the number of unanswered questions per patient over the total number of questions per visit and time to complete the PROMs; (5.2) number of outcome measures missing over the total number of measures per participant and visit; and (5.3) time to complete PROMs, muscle activity and kinematic outcome measures and the total time required to complete all assessment procedures.

Participants' adherence to the prescribed 4 h of taVNS per day was electronically logged by the app on their smartphones. Treatment was deemed compliant when at least 80% of participants used the stimulator for a minimum of 2 h each day. Pain intensity was evaluated using the GCPS questionnaire [24]. The incidence of significant adverse events (e.g., life‐threatening incidents, permanent harm or sequela, or hospitalisation) potentially related to the intervention was assessed during each visit.

#### 2.4.2 Secondary Outcomes

Various PROMs (PHQ‐9 [26], GAD‐7 [27], PHQ‐15 [28], and OHIP‐G14 [29]) were used to assess participants' personal experiences during the trial. Additionally, the pain‐free mouth‐opening item from the DC/TMD examination was recorded. The mandibular range of motion was evaluated as maximum mouth opening (MMO), and the effect of taVNS on EMG activity of the main chewing muscles (masseter and temporalis anterior) was recorded using maximum volumetric contraction (MVC).

All primary and secondary outcome measures were evaluated at T0, T1, and T2.

### 2.5 Data Logging

With the participant's consent, a smartphone app compatible with Android and iOS was installed and connected via Bluetooth to the stimulator. The app tracked participants' daily stimulation time and average intensity to monitor compliance. Additionally, it provided participants with a convenient way to keep track of stimulation sessions, as the stimulation was set to occur for 4 h each day. After the 4‐week stimulation period, the protocol data was exported and analysed to determine the average daily stimulation time and intensity.

### 2.6 Sample Size Calculation

This study was designed to detect a large effect on pain intensity [30], assuming a moderate correlation among repeated measures (*r* ≥ 0.5), an alpha level of 5%, and a power of 95%. Based on those considerations, a sample size of 18 participants was required to detect a significant interaction between treatment allocation (stimulation vs. sham) and time points (visits T0, T1, T2) (G*Power 3.1.9.7) [31]. Assuming a drop‐out rate of 10%, a sample of 20 participants was initially recruited, 10 in each of the study groups.

### 2.7 Statistical Analysis

A 2 × 3 factorial design was used with factors group (stimulation, sham) and timepoint (Visits T0, T1, and T2) to determine the main and interaction effects on primary and secondary outcomes, which were statistically analysed via mixed‐measures ANOVA as implemented in SPSS 26. In cases of violations of the sphericity assumption, degrees of freedom for the within‐subject factor time point were adjusted with the Greenhouse–Geisser method. Global effects were determined through post hoc comparisons between individual conditions (i.e., per group or time point). The level of significance was set at *p* < 0.05. Bonferroni correction was used for multiple comparisons when appropriate. Estimates of per‐group treatment effect were calculated as mean and standard deviation (SD). These analyses provided a treatment effect estimate on each outcome measure. Effect size was calculated using partial eta‐squared values (η^2^). For result interpretation, a small effect corresponded to a η^2^ ≈0.01, a medium effect to ≈0.06, and a large effect to ≈0.14.

## 3 Results

### 3.1 Participants' Recruitment and Characteristics

**FIGURE 2:**
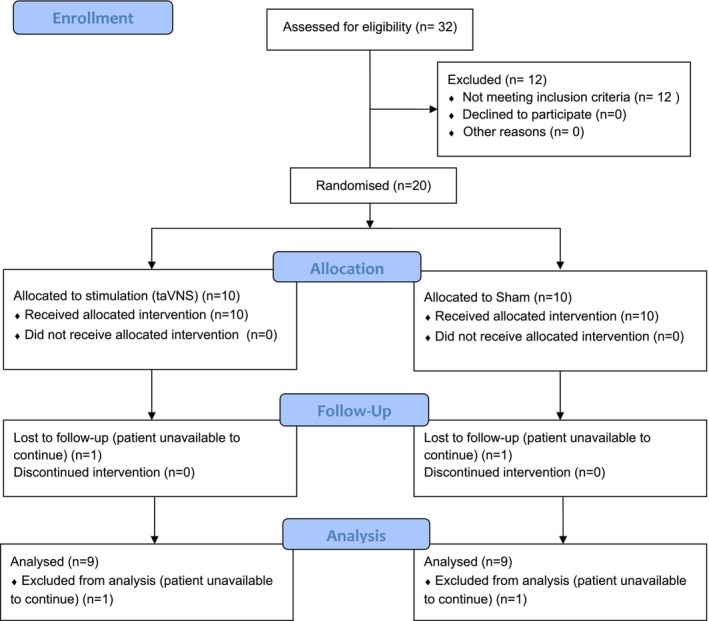
Flow‐Diagram of screening, enrollment, intervention allocation, follow‐up, and assessment of the effects of taVNS compared to sham this.

Thirty‐two participants were screened between March 2023 and March 2024 at the Department of Prosthodontics, University Medical Center Düsseldorf. A total of 20 participants with chronic pain associated with TMD (Grade III or IV of the Graduation of Chronic Pain Scale [GCPS] according to von Korff [24]) met the eligibility criteria and were randomised to taVNS (*n* = 10) or sham (*n* = 10). During follow‐up, one participant from each group was lost due to unavailability to continue the study for personal reasons, resulting in nine participants in each group being included for analysis (Figure 2). A common feature of both participants lost to follow‐up was the presence of Grade IV (GCPS). Participants' mean age was 44.5 ± 17.2 years, predominately female (1 male, 19 female), with a slightly higher frequency of Korff grading IV among those in the sham versus the taVNS group (Table 2).

**TABLE 2:** Participants' demographics according to study arm.

Variable	taVNS (*n* = 10)	Sham (*n* = 10)
Age		
18–29	3 (30%)	2 (20%)
30–39	1 (10%)	3 (30%)
> 40	6 (60%)	5 (50%)
Sex		
Male	1 (10%)	0 (0%)
Female	9 (90%)	10 (100%)
Korff‐Grading		
III	5 (50%)	2 (20%)
IV	5 (50%)	8 (80%)
Number of prior visits to clinicians		
1–3	4 (40%)	1 (10%)
4–6	5 (50%)	7 (70%)
7–10	1 (10%)	2 (20%)
Number of previous therapies		
0–1	3 (30%)	0 (0%)
2	3 (30%)	7 (70%)
3–4	4 (40%)	3 (30%)

Abbreviation: taVNS: transcutaneous auricular vagus nerve stimulation.

### 3.2 Outcomes and Estimations

#### 3.2.1 Feasibility, Compliance, and Adverse Events

Twenty participants enrolled in the study in a year (on average, two participants per month), reaching 100% of the target recruitment goal. Out of the 20 participants, 18 (90%) completed all outcome measures across visits 0, 1, and 2, with a retention rate exceeding the 75% target. Randomisation was achieved in 100% of participants allocated to the intervention or control groups. Blinding utilising an identically‐looking non‐connected sham electrode proved successful in all participants. In addition, 100% of the PROMs were completed across all visits, with an average of approximately 60 min, ranging between 45 and 120 min, to fill out all questionnaires per visit. Compliance with muscle activity and kinematic outcome measures also achieved 100%, taking approximately 90 min, ranging between 60 and 120 min. The total time required to complete all assessment procedures, including questionnaires and muscle activity and kinematic assessments, was approximately 180 min per participant. Participants' adherence to taVNS was 83% (*n* = 15), with three (two in group verum, one in group sham) individuals missing one out of 30 days. No serious adverse events were recorded. No local manifestations of a skin irritation or discomfort were observed.

#### 3.2.2 Patient‐Reported Outcome Measures

##### 3.2.2.1 Pain Intensity (GCPS)

Participants receiving taVNS reported lower levels of pain intensity at all time points compared to those receiving sham. Participants assigned to taVNS experienced a small but potentially important reduction in pain intensity across time points, as compared to those receiving sham. However, this difference was not large enough for the time point by treatment group interaction to reach statistical significance (Table 3).

**TABLE 3:** Group statistics for patient‐reported outcome measures. F‐statistics, *p* values, and effect sizes show ANOVA results for the treatment group by time point interaction effect.

		taVNS		Sham				
Outcome measure		Mean ± SD	95% CI	Mean ± SD	95% CI	F‐statistic (df)	*p*	Partial Eta^2^
GCPS	T0	63.2 ± 6.1	58.6–67.9	72.6 ± 10.8	64.3–80.9	0.175 (1.48, 23.69)	0.775	0.011
	T1	42.5 ± 19.4	27.6–57.4	52.6 ± 14.2	41.6–63.5			
	T2	48.5 ± 23.7	30.3–66.7	62.9 ± 11.6	53.9–71.8			
PHQ‐9	T0	13.4 ± 5.5	9.2–17.7	9.4 ± 7.3	3.9–15.0	0.536 (2, 32)	0.590	0.032
	T1	7.8 ± 4.8	4.1–11.5	6.1 ± 2.9	3.9–8.3			
	T2	10.9 ± 7.6	5.0–16.8	7.1 ± 5.2	3.2–11.1			
GAD‐7	T0	12.3 ± 4.6	8.8–15.8	6.8 ± 4.3	3.5–10.1	0.255 (2, 32)	0.776	0.016
	T1	8.3 ± 5.4	4.2–12.5	4.2 ± 2.6	2.2–6.2			
	T2	10.6 ± 6.7	5.4–15.7	5.1 ± 4.1	2.0–8.3			
PHQ‐15	T0	11.7 ± 5.0	7.8–15.5	7.8 ± 4.6	4.3–11.3	0.223 (2, 32)	0.802	0.014
	T1	9.1 ± 4.3	5.8–12.4	6 ± 3.7	3.12–8.8			
	T2	10.2 ± 5.9	5.7–14.8	6 ± 3.3	3.5–8.6			
OHIP‐14	T0	24.6 ± 12.1	15.2–33.9	13.3 ± 13.1	3.3–23.4	2.091 (2, 32)	0.140	0.116
	T1	14.0 ± 7.5	8.2–19.8	9.7 ± 10.0	2.0–17.3			
	T2	21.9 ± 14.8	10.5–33.2	10.6 ± 9.7	3.1–18.0			
JFLS‐20	T0	21.9 ± 12.3	12.5–31.3	17.4 ± 11.9	8.3–26.6	0.178 (1.39, 22.25)	0.758	0.011
	T1	15.1 ± 10.2	7.3–22.9	12.9 ± 12.2	3.5–22.2			
	T2	16.2 ± 11.5	7.4–25.1	14.2 ± 15.9	2.0–26.5			

Abbreviations: CI, Confidence interval; df, degrees of freedom; GAD‐7, Generalised Anxiety Disorders (higher score is worse); GCPS, Graduated Chronic Pain Scale (Pain intensity, higher score is worse); JFLS‐20, Jaw Functional Limitation Scale, Chewing ability (higher score is worse); OHIP‐14, Oral Health Impact Profile (Oral health‐related quality of life, higher score is worse); PHQ‐15, Patient Health Questionnaire (Severity of somatic symptoms, higher score is worse); PHQ‐9, Patient Health Questionnaire (Depression, higher score is worse); SD, Standard deviation.

##### 3.2.2.2 Depression (PHQ‐9)

At T1, both groups experienced a reduction in the PHQ‐9 score compared to T0. This was compatible with a change from moderate to mild depression, which potentially represents at least a small to medium global treatment effect. At T2, both arms experienced a trivial increase in their PHQ‐9 score. Group differences in the treatment effect for the PHQ‐9 score were not statistically significant (Table 3).

##### 3.2.2.3 Generalised Anxiety Disorders (GAD‐7)

At T0, on average, participants in the taVNS group reported moderate anxiety, while those in the sham group exhibited mild anxiety. At T1, both groups experienced a significant reduction in their GAD‐7 score, corresponding to mild anxiety in the taVNS and minimal anxiety in the sham group. At T2, both groups experienced a trivial increase in the GAD‐7 score. Participants assigned to taVNS experienced a small but potentially important effect on anxiety compared with sham. However, this difference was not large enough for the time point by treatment group interaction to reach statistical significance (Table 3).

##### 3.2.2.4 Severity of Somatic Symptoms (PHQ‐15)

Participants in the taVNS group presented a change from medium (T0) to low symptom severity (T1). On the other hand, participants in the sham group did not experience a category change (low severity at T0 and T1) in their PHQ‐15 score. No significant changes were observed in either group from T1 to T2. Participants assigned to taVNS experienced a small but potentially important effect on somatic symptoms compared with participants allocated to sham. However, this difference was not large enough for the time point by treatment group interaction to reach statistical significance (Table 3).

##### 3.2.2.5 Oral Health‐Related Quality of Life (OHIP‐G14)

Participants in the taVNS group experienced a change from a moderate (T0) to a mild oral health‐related quality of life (OHRQL) impact at T1 and T2. However, the OHRQL score slightly increased from T1 to T2. Participants in the sham group did not experience a category change (i.e., remained a mild impact on OHRQL at T1 and T2 compared to T0) in their OHRQL status. Participants assigned to taVNS experienced a potentially large effect on their OHRQL score compared with participants allocated to sham. However, this difference was not statistically significant (Table 3).

##### 3.2.2.6 Chewing Ability (JFLS‐20)

Overall, all participants exhibited low levels of chewing ability disturbance regardless of the time point assessed. Participants assigned to taVNS experienced a small but potentially important effect on the JFLS‐20 score compared with participants allocated to sham. However, this difference was not statistically significant (Table 3).

#### 3.2.3 Muscle Activity and Kinematics

##### 3.2.3.1 Maximum Volumetric Contraction (MVC)

Across all muscles, muscle side, and timepoints, participants receiving taVNS show higher means of MVC compared with those receiving sham. Participants assigned to taVNS experienced a small but potentially important effect on MVC compared to sham. However, these differences were not statistically significant (Table 4).

**TABLE 4:** Group statistics for muscle activity and kinematics. F‐statistics, *p* values, and effect sizes show ANOVA results for the treatment group by time point interaction effect.

		taVNS		Sham				
Outcome measure		Mean ± SD	95% CI	Mean ± SD	95% CI	F‐statistic (df)	*p*	Partial Eta^2^
MVC (Masseter right)	T0	389.2 ± 229.1	213.1–565.3	351.8 ± 264.0	148.9–554.7	0.797 (2, 32)	0.459	0.047
	T1	385.6 ± 250.3	193.2–578.0	370.1 ± 265.9	165.7–574.5			
	T2	403.2 ± 289.5	173.7–632.6	316.8 ± 209.8	155.6–478.1			
MVC (Masseter left)	T0	353.5 ± 219.5	184.8–522.2	348.6 ± 251.0	155.7–541.5	0.065 (2, 32)	0.937	0.004
	T1	403.4 ± 215.3	237.9–569.0	405.3 ± 309.1	167.7–643.0			
	T2	430.4 ± 285.3	211.1–649.7	404.4 ± 229.0	228.4–580.5			
MVC (Temporalis right)	T0	286.3 ± 163.2	160.8–411.8	259.2 ± 138.1	153.1–365.3	1.675 (1.16, 18.49)	0.214	0.095
	T1	335.7 ± 182.9	195.1–476.3	242.0 ± 128.3	143.5–340.6			
	T2	294.3 ± 153.0	176.7–411.9	278.3 ± 198.5	125.7–430.8			
MVC (Temporalis left)	T0	266.6 ± 154.8	147.7–385.6	210.5 ± 141.3	102.0–319.2	2.164 (2, 32)	0.131	0.119
	T1	295.4 ± 164.2	169.2–421.6	207.2 ± 91.9	136.6–277.7			
	T2	254.3 ± 119.3	162.6–346.0	236.7 ± 153.4	118.8–354.6			
MMO	T0	52.3 ± 4.9	48.6–56.1	54.9 ± 7.7	48.9–60.9	0.262 (2, 32)	0.771	0.016
	T1	56.1 ± 5.3	52.0–60.2	57.5 ± 3.6	54.7–60.3			
	T2	53.5 ± 6.8	48.3–58.6	53.9 ± 6.1	49.2–58.5			
PFJO	T0	27.3 ± 5.9	22.8–31.9	29.1 ± 5.4	25.0–33.3	0.418 (2, 32)	0.662	0.025
	T1	32.7 ± 9.9	25.0–40.3	36.7 ± 6.4	31.7–41.6			
	T2	32.8 ± 5.4	28.6–37.0	33.0 ± 9.9	25.4–40.6			

Abbreviations: CI, Confidence interval; df, degrees of freedom; MMO, Maximum mouth opening incisal (Kinematics, mm); MVC, Maximum volumetric contraction (Muscle activity, μV); PFJO, Pain free jaw opening incisal (Kinematics, mm); SD, Standard deviation.

##### 3.2.3.2 Maximum Mouth Opening (MMO)—Incisal

In both groups under investigation, MMO remained similar across timepoints. Participants assigned to taVNS experienced a small but potentially important effect compared with sham; however, this difference was not large enough for the time point by treatment group interaction to reach statistical significance (Table 4).

##### 3.2.3.3 Pain Free Jaw Opening—Incisal

In both groups under investigation, pain‐free jaw opening remained similar at T0, T1, and T2. Participants assigned to taVNS experienced a small but potentially important effect compared with sham; however, this difference was not statistically significant (Table 4).

## 4 Discussion

The study findings showed that assessing the impact of taVNS in participants with chronic pain associated with TMD using a RCT design is feasible. Engagement was high, which included achieving 100% of the recruitment target, 83% adherence, 90% retention, and no serious adverse events were reported. A large effect of taVNS on OHRQL and at least a small but potentially important effect on several clinical outcomes was observed, though they were not statistically significant.

The establishment of a sham‐controlled group in taVNS studies remains challenging. Earlobe stimulation, although it is not vagally innervated [32], can activate nearby muscles [33, 34] and may alert participants to their placebo status. Low‐frequency stimulation (e.g., 1 Hz) is also unreliable, as it may still have therapeutic effects [35, 36]. The tVNS‐L device used has fixed parameters, while the adjustable tVNS‐R device lacks CE certification, which limits its use in clinical trials. Future trials could explore a second stimulation group with lower parameters to refine the treatment protocol.

Participants associated with Grade IV (GCPS) were found to be challenging to follow up. This may be attributed to the severity of their chronic pain, which causes major disability and restriction of activities of daily living. To reduce loss of follow up, a run‐in period [37] with a sham device can assess the participants' adherence. Mitigation strategies (e.g., incentives to attend visits, flexible schedule, and adaptive outcome measure techniques like telephone or instant messages) can also be considered, especially for those starting with or changing to a Grade IV.

A placebo effect varies based on participant amenability, mental state, and type of placebo used [38]. Evidence suggests that participants with anxiety disorders are particularly sensitive to placebo effects [38], and those with greater therapeutic experience often have higher expectations, leading to stronger placebo responses [39]. In this study, improvements in TMD symptoms may also relate to a “care effect” from lengthy study appointments, wellbeing journaling, and stress reduction, as participants often slowed down during the 4 h of daily device use at home. To account for the observed placebo and care effect, future trials should include a sham device as control.

This pilot study has limitations. While participants were blinded to the treatment allocation, the assessors were not, introducing the potential for bias in outcome assessments. Future clinical trials should implement blinding of participants and outcome adjudicators to enhance the rigour of the findings. Additionally, the app designed to record stimulation intensity and duration had some issues with a reliable connection to the stimulator, necessitating the use of hand‐written diaries as a back‐up. While this approach ensured data collection, it may compromise the accuracy and reliability of the data. Another limitation of this pilot trial is that the results only reflect short‐term effects. Future studies should investigate the long‐term efficacy of taVNS to understand its sustained impact on chronic pain management. The strength of the present pilot trial lies in the innovative approach exploring taVNS as a promising non‐pharmacological treatment for patients with chronic TMD symptoms that is both safe and well‐tolerated [9]. An additional advantage of taVNS is its compatibility with other therapeutic modalities, such as splint therapy, physical therapy, or pharmacological treatments. The ear pod‐style design allows patients to maintain their daily routines while undergoing stimulation [35]. The present study included a small and predominately female sample (19 females and 1 male), which may limit the generalizability of the results. However, this gender distribution reflects the clinical and epidemiological features of TMD. Studies have consistently shown that female‐to‐male ratios range from 2:1 to 6:1 [40, 41]. Future trials should recruit larger and more diverse samples to gain a broader understanding of taVNS treatment effects across different populations. Although the number of participants for target recruitment was met, a following trial should be conducted as a multicenter study. Since our preliminary evidence suggests that the magnitude of the effect of taVNS may be small but important to moderate, sample size calculations for the definitive trial should be modified to consider a target effect that is at least small but important (e.g., minimal important difference [42]) rather than a large effect. The 4‐week stimulation period aligns with taVNS trials in chronic pain conditions, such as migraine [43, 44], where symptom improvements have been observed within this timeframe. However, longer intervention durations may be necessary to assess the sustainability of these effects.

## 5 Conclusion

This pilot trial confirms the feasibility of taVNS for chronic TMD, with high recruitment, retention, and compliance rates. A large effect of taVNS was detected in OHRQL and at least a small but important effect on participants' pain intensity, anxiety, depression, severity of somatic symptoms, chewing ability, muscle activity, and kinematics. These promising estimations are hypothetical since no outcomes reached statistical significance. The intervention was safe and well‐tolerated, with no adverse events reported.

## Author Contributions

Mortimer Gierthmuehlen and Petra C. Gierthmuehlen had the idea of the study and contributed to its design. Lea S. Prott is the principal investigator and wrote the original draft of the manuscript. Lea S. Prott, Vanessa Kaldenhoven and Alfons Hugger were responsible for the patient recruitment, examinations, and the organisation of treatments. Robert Langner conducted the data analysis. All authors reviewed and approved the final manuscript.

## Ethics Statement

This trial was approved by the Ethics Commission of the University Hospital Düsseldorf (Reference number: 2022‐1889) on July 1, 2022. Accepted version of the study protocol is 1.3.

## Consent

At the initial study visit, participants signed an informed consent document explaining the study's purpose, procedures, risk, benefits, and were given the opportunity to ask any questions. The study was designed according to Good Clinical Practice (GCP), the principles of the Declaration of Helsinki and standards for professional conduct. This study manuscript was written following the reporting standards of the CONSORT extension to randomised pilot and feasibility trials [45].

## Conflicts of Interest

M.G. is founder and advisor of the Neuroloop GmbH. This start‐up develops an invasive vagus nerve stimulator against arterial hypertension. The company has no link to this study, and the topic (non‐invasive VNS, pain) does not interfere with the company's focus (invasive VNS, hypertension).

## Data Availability

The data that support the findings of this study are available from the corresponding author upon reasonable request.
